# Frequency Dependent Electrical Stimulation of PFC and ACC for Acute Pain Treatment in Rats

**DOI:** 10.3389/fpain.2021.728045

**Published:** 2021-08-27

**Authors:** Yaling Liu, Helen Xu, Guanghao Sun, Bharat Vemulapalli, Hyun Jung Jee, Qiaosheng Zhang, Jing Wang

**Affiliations:** ^1^Department of Anesthesiology, Perioperative Care and Pain Medicine, New York University School of Medicine, New York, NY, United States; ^2^Interdisciplinary Pain Research Program, New York University Langone Health, New York, NY, United States; ^3^Department of Psychiatry, New York University School of Medicine, New York, NY, United States; ^4^Department of Neuroscience & Physiology, New York University School of Medicine, New York, NY, United States; ^5^Neuroscience Institute, New York University School of Medicine, New York, NY, United States

**Keywords:** pain, electrical stimulation, neuromodulation, prefrontal cortex, anterior cingulate cortex

## Abstract

As pain consists of both sensory and affective components, its management by pharmaceutical agents remains difficult. Alternative forms of neuromodulation, such as electrical stimulation, have been studied in recent years as potential pain treatment options. Although electrical stimulation of the brain has shown promise, more research into stimulation frequency and targets is required to support its clinical applications. Here, we studied the effect that stimulation frequency has on pain modulation in the prefrontal cortex (PFC) and the anterior cingulate cortex (ACC) in acute pain models in rats. We found that low-frequency stimulation in the prelimbic region of the PFC (PL-PFC) provides reduction of sensory and affective pain components. Meanwhile, high-frequency stimulation of the ACC, a region involved in processing pain affect, reduces pain aversive behaviors. Our results demonstrate that frequency-dependent neuromodulation of the PFC or ACC has the potential for pain modulation.

## Introduction

Pain has sensory and affective components, making it difficult to be managed entirely by pharmaceutical treatments. Meanwhile, reliance on opioid analgesics causes side effects such as addiction (1, 2). Non-pharmaceutical methods of pain relief, in the form of neuromodulation, can play an important role in the dual epidemic of chronic pain and opioid addiction. In recent years, electrical stimulation has emerged as a promising treatment option (3–6). However, further preclinical studies on stimulation targets and parameters are needed to fully support its clinical application.

The medial and dorsolateral prefrontal cortices (PFC) and the anterior cingulate cortex (ACC) play critical roles in acute and chronic pain modulation. Previous studies have shown that acute noxious stimuli could induce an increase in neuronal firing in those regions (7–10). The PFC is an important center for top-down regulation of sensory stimuli (11). Previous animal and clinical imaging studies have demonstrated synaptic changes in the PFC during acute and chronic pain states (12–18), and stimulation of components of the PFC can increase the latency of withdrawal and reduce aversive responses to noxious stimuli (19–23). The ACC, meanwhile, is involved particularly in the processing of the affective, or aversive, component of pain (24–28). Activity in this region has been shown to be elevated in response to or anticipation of noxious chemical, thermal, and mechanical stimuli (8, 9, 29–32). Activation of the ACC, in contrast to the PFC, enhances pain aversion (8, 33).

Recent preclinical studies have demonstrated that optogenetic activation of the PFC can lead to the decrease of both nociceptive and neuropathic pain (21–23, 34), whereas the activation of ACC powerfully enhances the affective component of pain (8, 33). However, due to the need for expression of a viral vector in the brain, optogenetic treatment is not currently feasible in most clinical settings. In contrast, electrical stimulation has been safely and effectively utilized in humans (35–46). A previous animal study showed that low-frequency 2-Hz electrical stimulation to the PFC can be used to enhance the endogenous pain-inhibitory function (47). However, to optimize this technology, further studies are needed to characterize the effects of a wide range of stimulation frequencies in cortical targets.

In this study, we investigated the effects of electrical stimulation of the prelimbic area of the prefrontal cortex (PL-PFC) or the ACC as therapeutic methods to treat acute pain in awake, freely moving rats. We found that low-frequency electrical stimulation of the PL-PFC can inhibit both sensory and affective components of pain, whereas high-frequency electrical stimulation of the ACC can reduce the aversive components of pain. These results demonstrate the potential for cortical electrical stimulation in the treatment of pain.

## Materials and Methods

### Animals

All procedures were carried out in accordance with the guidance of the Institutional Animal Care and Use Committee (IACUC) of the New York University School of Medicine (NYUSOM) and were consistent with the “Guidelines for the Care and Use of NIH Laboratory Animals” to ensure minimal animal use and discomfort. Male Sprague-Dawley rats were purchased from Taconic Farm and kept at the vivarium facility in the NYU Langone Science Building, under controlled humidity, temperature and 12 h (6:30 a.m. to 6:30 p.m.) light-dark cycle. Food and water were available *ad libitum*. Before the start of the experiment, the animals arrived at the animal facility weighing 250–300 g and were given an average of 10 days to adjust to the new environment. A total of 10 rats were used for the experiments.

### Intracranial Electrode Implants

The intracranial bipolar electrodes were made by twisting two 75 μm diameter formvar insulated Stablohm 675 wires (California Fine Wire Corporation). At the stimulus end of the twisted electrodes, one wire was cut 0.4 mm shorter than the other, providing the distance for the flow of applied current, while allowing both ends to remain within the same cortical layer. Fracture surfaces of the stimulus ends were ensured to be bright and smooth. The other ends of the electrodes were not twisted and were insulated and coupled to a connector header (2163S-36-ND, Digi-Key). Each rat had a single PL-PFC electrode implantation on one side and an ACC implantation on the opposite side. Rats were anesthetized with isoflurane (1.5–2%). The skull was exposed and a 1.0 mm-diameter hole was drilled above the target region. After puncture of the dura mater with a 30 G needle, a stereotaxic apparatus was used to slowly lower the electrode into the PL-PFC or the ACC. The coordinates for the unilateral PL-PFC electrodes were as follows: anteroposterior (AP) +2.9 mm, mediolateral (ML) ± 1.6 mm, and dorsoventral (DV) −3.7 mm, with tips angled 17° toward the midline. The coordinates for the unilateral ACC electrodes were as follows: AP +3.0 mm, ML ± 0.6 mm, DV −1.5 mm, angled at 0° toward the midline. The electrodes were secured to the bone screws in the skull with dental cement. The rats were given 1 week for recovery from surgery before the experiments were performed.

After the experiments, the electrical lesions using direct current at 50 μA for 10 s were performed before the animal sacrifice. Rats were deeply anesthetized with isoflurane and perfused with cold phosphate-buffered saline (PBS) followed by p-formaldehyde (PFA). Twelve micrometers of thick brain sections were collected using Leica CM3050 s cryostat (Leica Biosystems). The slices were stained with cresyl violet and viewed using an Axio Zoom widefield microscope (Carl Zeiss). If the electrodes were implanted in the improper place, the animals were excluded from the further data analysis.

### Electrical Stimulation Protocols

All electrical stimulations were applied using Constant Current Stimulus Isolator (A365, World Precision Instruments). The stimulus sequence was triggered by Transistor-Transistor Logic (TTL) pulse generators (Tucker-Davis Technologies, TDT). The parameter of electrical stimulation was set at 2, 20, 40, 60, 80, and 120 Hz. A current amplitude of 20 μA was used during behavioral testing. Each pulse was given continuously for 5 s as a biphasic square wave with a stable duty cycle of 40%.

### Hargreaves Test (Plantar Test)

The Hargreaves test was performed to evaluate the response to acute thermal stimuli. A mobile radiant heat-emitting device with an aperture of 10 mm (37370 plantar test, Ugo Basile) was used to produce acute thermal stimulation of the plantar surface of the hind paw (21, 48). An IR intensity of 70 was used to provide noxious stimulation in the present study. The rats were placed in a plexiglass chamber over a Hargreaves glass table and allowed to habituate. An average of at least 5 trials was performed to measure the latency to paw withdrawal for each testing condition. In the DBS trials, the electrical stimulation was applied for a defined time window of 5 s, delivered simultaneously with the Hargreaves stimulation. This latency was automatically recorded, and an average latency across the trials was computed. Paw withdrawals resulting from locomotion or weight shifting were not counted, and the trials were repeated in such cases. Measurements were repeated at ~5-min inter-trial intervals. To prevent the association of a certain frequency with the effects of repeated thermal stimulation, we conducted the entire Hargreaves procedure using a random sequence of interspersed frequencies. Efforts were taken to minimize sensitivity to repeated thermal stimulation by limiting the number of electrical stimulation trials per frequency to three in each session. Intersession interval was least 4 h.

### Conditioned Place Aversion Assay

The conditioned place aversion (CPA) protocol used in this experiment was developed for testing the aversive response to acute noxious stimuli and has been described in recent literature (7, 8, 32). Each experiment was performed in a standard two connected chamber apparatus. In this CPA protocol, the preconditioning and testing phases (each consisting of 10 min), were performed before and after the conditioning phases, respectively. The conditioning was only conducted once per experiment, and during conditioning, rats spent 10 min in each treatment chamber. During the conditioning phase, the rats were given either a peripheral pain stimulus (PP) (or no pain stimulus (NS) as control), paired with or without electrical stimulation of the cortex. Each PP was administered at 30 s intervals using a 27 G needle to the middle of the rat's hind paw contralateral to the stimulating electrode. To prevent chamber orientation from becoming a confounding factor in the animals' aversive response, the electrical stimulus and chamber pairings were always counterbalanced. During the testing phase, the animals were given neither peripheral nor electrical stimulus, and had access to move freely between the chambers. The movements of rats in each chamber were automatically recorded by a camera and were analyzed with the AnyMaze software to determine the total time spent in each chamber. As compared with the baseline, decreased time spent in a chamber during the testing phase indicates avoidance (aversion) of that chamber. A CPA score was computed by subtracting the time spent in the more noxious chamber during the testing phase from the time spent in that chamber during the pre-conditioning phase. Distinct groups of animals were used for the control (no or sham stimulation) and treatment (electrical stimulation) groups in the CPA tests.

### Statistical Analysis

Results of behavioral experiments were given as mean ± s.e.m. A one-way ANOVA, Tukey's multiple comparisons test with repeated measures was used to analyze the results from the Hargreaves test. For the CPA test, a two-tailed paired Student's *t*-test was used to compare the time spent in each treatment chamber before and after conditioning (i.e., pre-conditioning vs. testing phase for each chamber). A two-tailed unpaired Student's *t*-test was used to compare the CPA scores under various conditions. For the locomotion distance traveled test, a one-way ANOVA, Tukey's multiple comparisons test with repeated measures was used. For all tests, a *p* < 0.05 was considered statistically significant. All data were analyzed using the GraphPad Prism Version 9 software (GraphPad).

## Results

### Frequency Dependent Anti-nociceptive Effects of Electrical Stimulation of the PL-PFC

**Figure 1:**
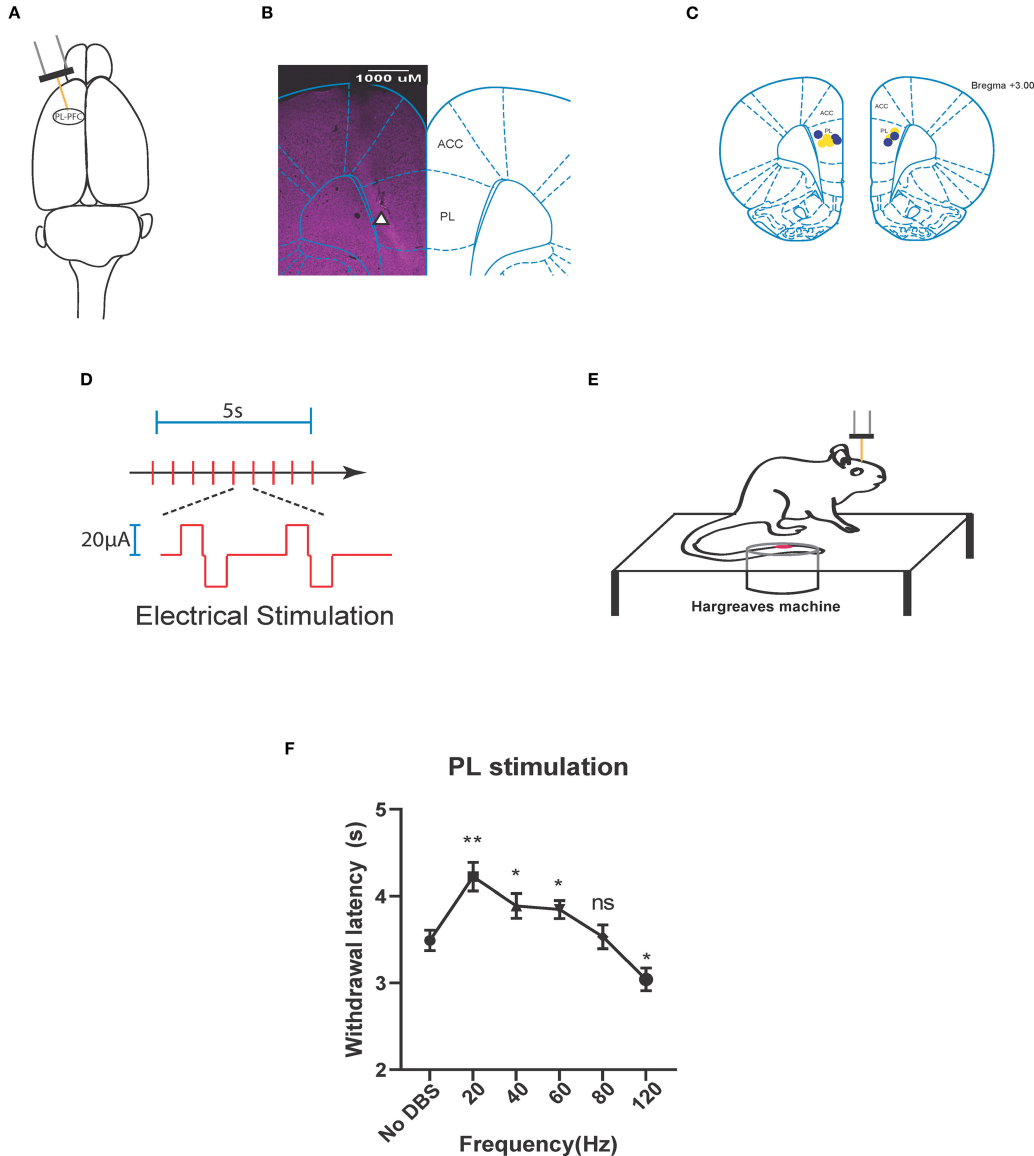
Neurostimulation of the PL-PFC alleviates acute thermal pain at low frequencies and increases it at high frequencies. **(A)** Stimulating electrode (orange) implanted in the left pre-limbic prefrontal cortex (PL-PFC). **(B)** Histology verifying the implantation of the electrode in the PL-PFC. White triangle indicates location of the electrode. **(C)** Schematic showing the intracranial electrode sites in the PL-PFC. Yellow dots denote the CPA control group implantations. Blue dots denote the CPA experimental group implantations. **(D)** Each red vertical line indicates administration of the electrical stimulation, given as a biphasic square wave. The area above the axis denotes an active stimulus phase. This is followed by a brief latency, then an active charge-balancing phase, represented by the area below the axis. **(E)** Schematic of the Hargreaves test (IR 70). Thermal stimulation from the infrared (IR) emitter is applied to the rat's hind paw, contralateral to the electrode implanted within the PL-PFC. Electrical stimulation is delivered to the PL-PFC concurrent with onset of the thermal stimulation. **(F)** Application of lower frequency (20, 40, and 60 Hz) electrical stimulation of the PL-PFC prolonged the withdrawal latency relative to that of the baseline, in which no DBS was given, whereas application of high frequency (120 Hz) decreased the withdrawal latency. Electrical stimulation of the PL at 80 Hz yielded no change in withdrawal latency relative to that of the baseline. *n* = 9 rats. No DBS vs. 20 Hz, ^**^P = 0.044; No DBS vs. 40 Hz, ^*^P = 0.0491; No DBS vs. 60 Hz, ^*^P = 0.0456; No DBS vs. 80 Hz, *P* = 0.9991; No DBS vs. 120 Hz, ^*^P = 0.0436; one-way ANOVA, Tukey's multiple comparisons test with repeated measures. Data are presented as mean ± s.e.m.

We provided electrical neurostimulation at various frequencies (20, 40, 60, 80, 120 Hz) to determine the effects of stimulation frequency on prefrontal control of acute thermal pain. We inserted stimulating electrodes selectively into the PL-PFC region of naïve rodents (Figures 1A–C). We provided electrical stimulation in the form of a biphasic square wave (Figure 1D) in a range of frequencies, during simultaneous administration of a noxious thermal stimulus during a Hargreaves test (Figure 1E). We delivered our electrical stimulation at an intensity of 20 μA, as this intensity has been shown to be used safely in a previous experiment in which the PL-PFC was stimulated at low-frequencies to modulate acute thermal pain (47). We found that stimulation at 20–60 Hz provided pain relief relative to the control (sham stimulation) condition, where no DBS was given. Interestingly, at very high frequency (120 Hz), electrical stimulation increased acute nociceptive withdrawals, suggesting decreased prefrontal control of pain (Figure 1F). These results indicate that prefrontal control of pain can be enhanced by low-frequency neurostimulation and may in fact be decreased by high-frequency neurostimulation.

A previous study has shown that PL-PFC electrical stimulation at 2 Hz, which is within the physiological range, could provide pain relief (47). Here, we compared stimulation at 2 Hz with our lowest stimulating frequency of 20 Hz. We conducted a Hargreaves test comparing the paw withdrawal latencies in response to unilateral electrical stimulation of the PL-PFC at 2 and 20 Hz (Supplementary Figure 1A). We found that while both 2 and 20-Hz electrical stimulations to the PL-PFC increased the withdrawal latency relative to that of the sham stimulation condition, the 20-Hz electrical stimulation was even more effective (Supplementary Figure 1B).

### Frequency Dependent Anti-aversive Effects of Electrical Stimulation of the PL-PFC

**Figure 2:**
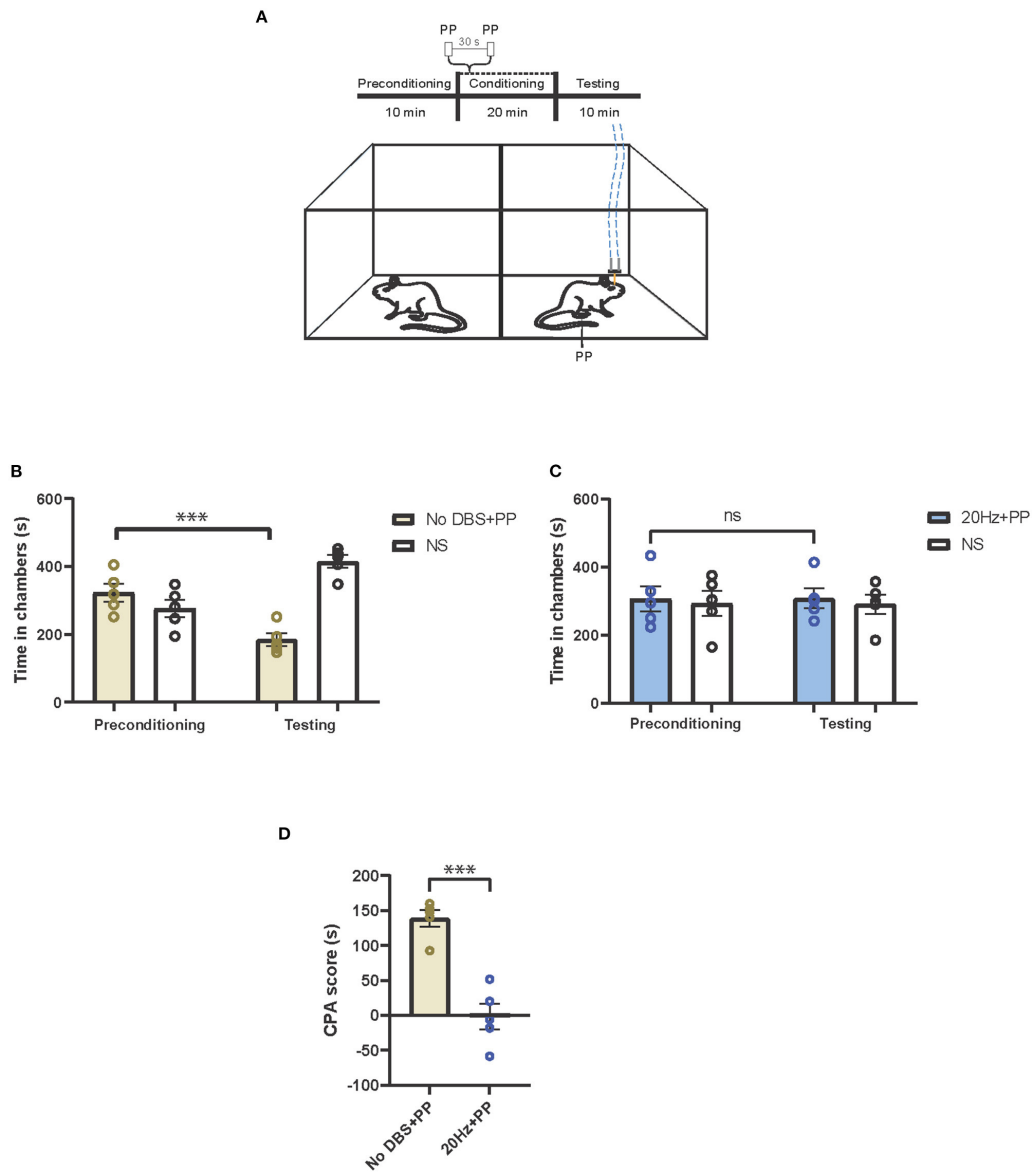
Low-frequency neurostimulation of the PL-PFC decreases pain aversive response. **(A)** Schematic of the conditioned place aversion (CPA) assay to test the effect of PL-PFC stimulation on pain aversion in response to a noxious mechanical stimulus (pinprick, PP). One of the chambers was paired with PP applied to the hind paws 30 s apart, in addition to 20-Hz (or sham) stimulation of the PL-PFC, whereas the opposite chamber was paired with no peripheral stimulation (NS) and no electrical stimulation of the PL-PFC. The dashed blue line denotes electrical stimulation. **(B)** After conditioning, rats developed an aversion to the treatment chamber associated with PP paired with sham stimulation of the PL-PFC. *n* = 5; ^***^P = 0.0003, paired *t-*test. Data are presented as mean ± s.e.m. **(C)** After conditioning, rats did not develop an aversion to the chamber associated with PP paired with 20-Hz stimulation of the PL-PFC. *n* = 5; *P* = 0.9094, paired *t*-test. Data are presented as mean ± s.e.m. **(D)** CPA scores for rats given noxious mechanical stimulation (PP) paired with sham electrical stimulation (yellow) compared to rats that were given PP paired with 20-Hz electrical stimulation (blue) to the PL-PFC. *n* = 5; ^***^P = 0.0002, unpaired *t*-test. Data are presented as mean ± s.e.m.

Next, we tested if electrical stimulation to the PL-PFC could reduce the aversive response to pain using a classic two-chamber conditioned place avoidance assay (CPA) in rats (Figure 2A) (8, 22). In this assay, during the preconditioning phase, rats were allowed to move freely between the two chambers. Next, during conditioning, one chamber was paired with a noxious stimulus, whereas the opposite chamber was not. Finally, during the testing phase, the peripheral stimulus was removed, and rats were allowed to freely move between both chambers. Previous results indicate that during the testing phase, rats avoided the chamber paired with the noxious stimulus due to the associated pain-aversive experience (8, 32). In our control experiment, one chamber was paired with a peripheral pain stimulus (PP) and sham PL-PFC stimulation (No DBS), and the other chamber was paired with no pain stimulus (NS). The rats avoided the chamber with the peripheral pain stimulus, suggesting that the pain stimulus indeed produced an aversive pain response that is not alleviated by sham stimulation alone (Figure 2B). Next, we repeated the CPA assay by administering no pain stimulus (NS) in one chamber, and 20-Hz electrical stimulation paired with peripheral pain stimulus (PP) in the opposite chamber. We found that the rats did not exhibit an avoidance for either chamber (Figure 2C), suggesting that the 20-Hz electrical stimulation decreases the aversive behavior in response to the peripheral pain stimulus. To quantitate these results, we calculated a CPA score by subtracting the amount of time the rat spent in the pain stimulus-paired chamber during the testing phase from the amount of time it spent in the preconditioning phase. Twenty hertz electrical stimulation of the PL-PFC reduced the CPA score relative to sham stimulation, confirming a reduction of pain aversive response (Figure 2D).

Having determined that electrical stimulation of the PL-PFC at physiological levels (2 Hz) could produce partial anti-nociceptive effects, we compared the anti-aversive response of this frequency with that of 20-Hz electrical stimulation. In our CPA assay, we delivered peripheral pain stimulus (PP) to both chambers, pairing one chamber with 2-Hz electrical stimulation and the other with 20-Hz stimulation (Supplementary Figure 1D). The rats exhibited an avoidance for the chamber paired with the 2-Hz stimulation, suggesting that electrical stimulation at 20-Hz produced greater anti-aversive effects (Supplementary Figure 1D).

**Figure 3:**
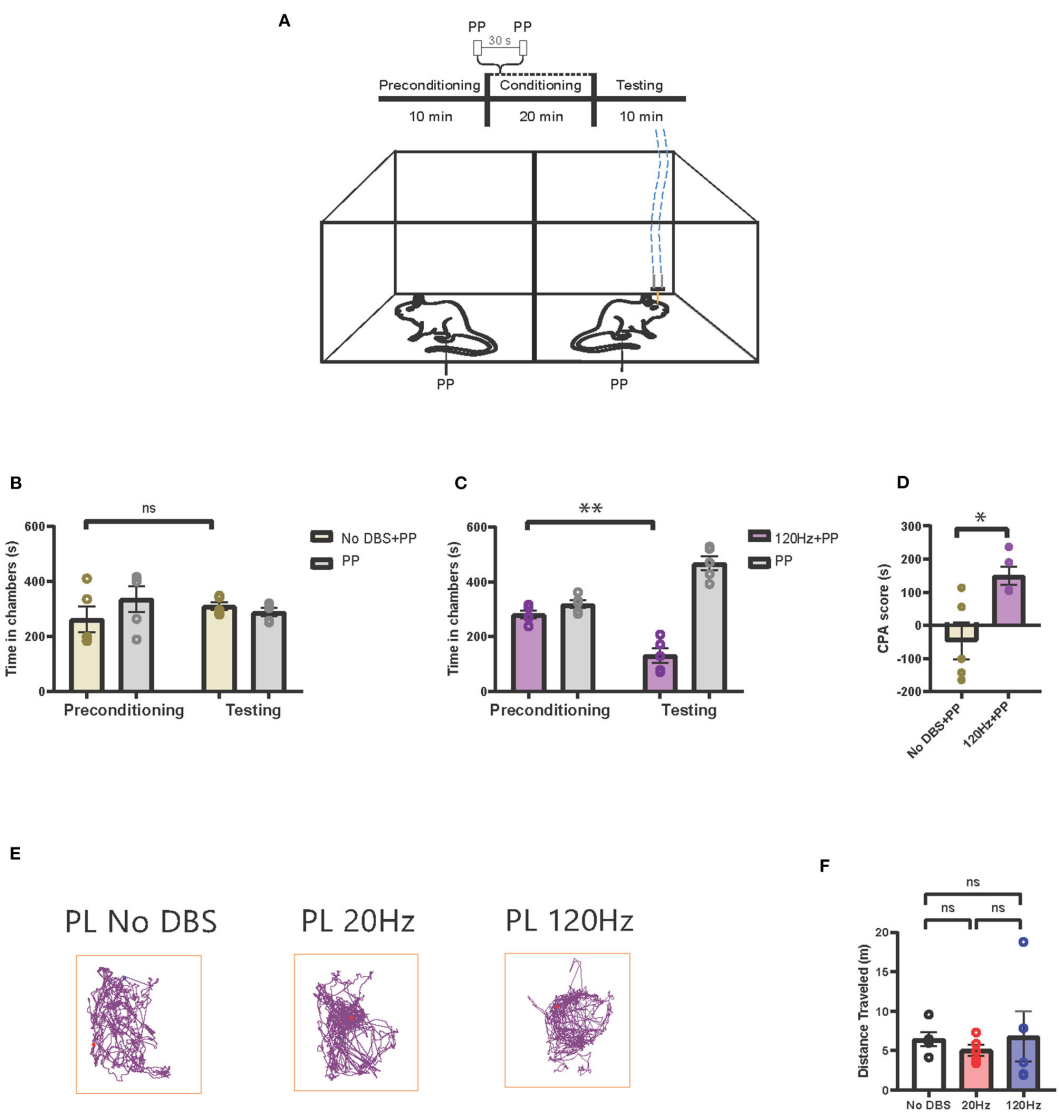
High-frequency neurostimulation of the PL-PFC increases pain aversive response. **(A)** Schematic of the CPA assay to test pain aversion under high-frequency stimulation of PL-PFC. Aversive response was triggered by a noxious mechanical stimulus (PP) applied to the hind paws in both chambers 30 s apart. In the control chamber, the mechanical stimulus was not paired with electrical stimulation; in the treatment chamber, the mechanical stimulus was paired with 120-Hz electrical stimulation to the PL-PFC, denoted by the blue dashed line. **(B)** After conditioning, rats receiving noxious mechanical stimulation (PP) paired with sham electrical stimulation to the PL-PFC did not develop an aversion to the treatment chamber. *n* = 5; *P* = 0.4390, paired *t*-test. Data are presented as mean ± s.e.m. **(C)** After conditioning, rats receiving noxious mechanical stimulation (PP) paired with 120-Hz electrical stimulation to the PL-PFC developed an aversion to the treatment chamber. *n* = 5; ^**^P = 0.0053, paired *t*-test. Data are presented as mean ± s.e.m. **(D)** CPA scores for rats given noxious mechanical stimulation (PP) paired with sham electrical stimulation (yellow) compared to rats that were given PP paired with 120-Hz electrical stimulation (purple) to the PL-PFC. *n* = 5; ^*^P = 0.0129, unpaired *t*-test. Data are presented as mean ± s.e.m. **(E)** Locomotion of the rat within a chamber recorded by AnyMaze when given electrical stimulation at different frequencies (No DBS, 20 and 120 Hz) to the PL-PFC. The purple line denotes the path taken; the red dot marks the origin. The frequency of the electrical stimulation administered had no noticeable effects on locomotion. **(F)** The frequency of electrical stimulation to the PL-PFC had no significant effect on locomotion. *n* = 5; No DBS vs. 20 Hz, *P* = 0.3431; No DBS vs. 120 Hz, *P* = 0.9949; 20 Hz vs. 120 Hz, *P* = 0.8874; one-way ANOVA, Tukey's multiple comparisons test with repeated measures. Data are presented as mean ± s.e.m.

We then tested how high-frequency electrical stimulation of the PL-PFC affected the pain aversive response. In this CPA assay, we administered peripheral pain stimulus to both chambers, pairing one of the chambers with electrical stimulation (Figure 3A). If high-frequency PL-PFC stimulation increases the aversive value of the noxious stimulus, it should cause the rat to avoid the chamber associated with this treatment. For our control experiment, we used sham electrical stimulation. As there was no difference in time spent in the sham stimulation chamber during testing and preconditioning phases, we confirmed that sham stimulation did not impact pain aversive response (Figure 3B). Next, we repeated this CPA assay for the treatment condition, replacing the sham stimulation with 120-Hz high-frequency electrical stimulation during the conditioning phase. During the testing phase, rats avoided the chamber associated with 120-Hz electrical stimulation combined with the peripheral pain stimulus (Figure 3C). To quantitate these results, we calculated the CPA scores. High-frequency electrical stimulation of the PL-PFC increased the CPA score relative to sham stimulation, suggesting that high-frequency electrical stimulation to the PL-PFC increases the aversive response to pain (Figure 3D). To demonstrate that electrical stimulation in the PL-PFC produces no gross side effects, we measured the locomotion of the rats under low-, high-, and sham stimulation conditions. We found that neither low- nor high-frequency stimulation of the PL-PFC altered gross locomotive behavior (Figures 3E,F).

Next, to confirm our findings, we directly compared the effects of the low- (20 Hz) and high- (120 Hz) frequency PL-PFC stimulation on the aversive response to peripheral pain stimulus. In this assay, peripheral pain stimulus was administered in both chambers. The acute pain stimulus was paired with 20-Hz electrical stimulation in one chamber and 120-Hz stimulation in the opposite chamber (Supplementary Figure 1E). The results were consistent with our earlier data (Figures 2C, 3C), as we found that the rats avoided the chamber paired with 120-Hz electrical stimulation (Supplementary Figure 1F).

Finally, we conducted a CPA assay to verify that the aversive response to the 120-Hz electrical stimulation to the PL-PFC was specific to pain. We conducted a CPA assay where one chamber received no peripheral pain stimulus (NS) paired with 120-Hz electrical stimulation, and the other chamber received neither peripheral nor electrical stimulation (Supplementary Figure 1G). In the absence of a pain stimulus, there was no significant difference between the aversive response in the two chambers, suggesting that the aversion induced by 120-Hz electrical stimulation is pain specific (Supplementary Figure 1H).

### Electrical Stimulation of the ACC Increases Pain Aversive Response at Lower Frequencies and Reduces It at Higher Frequencies

**Figure 4:**
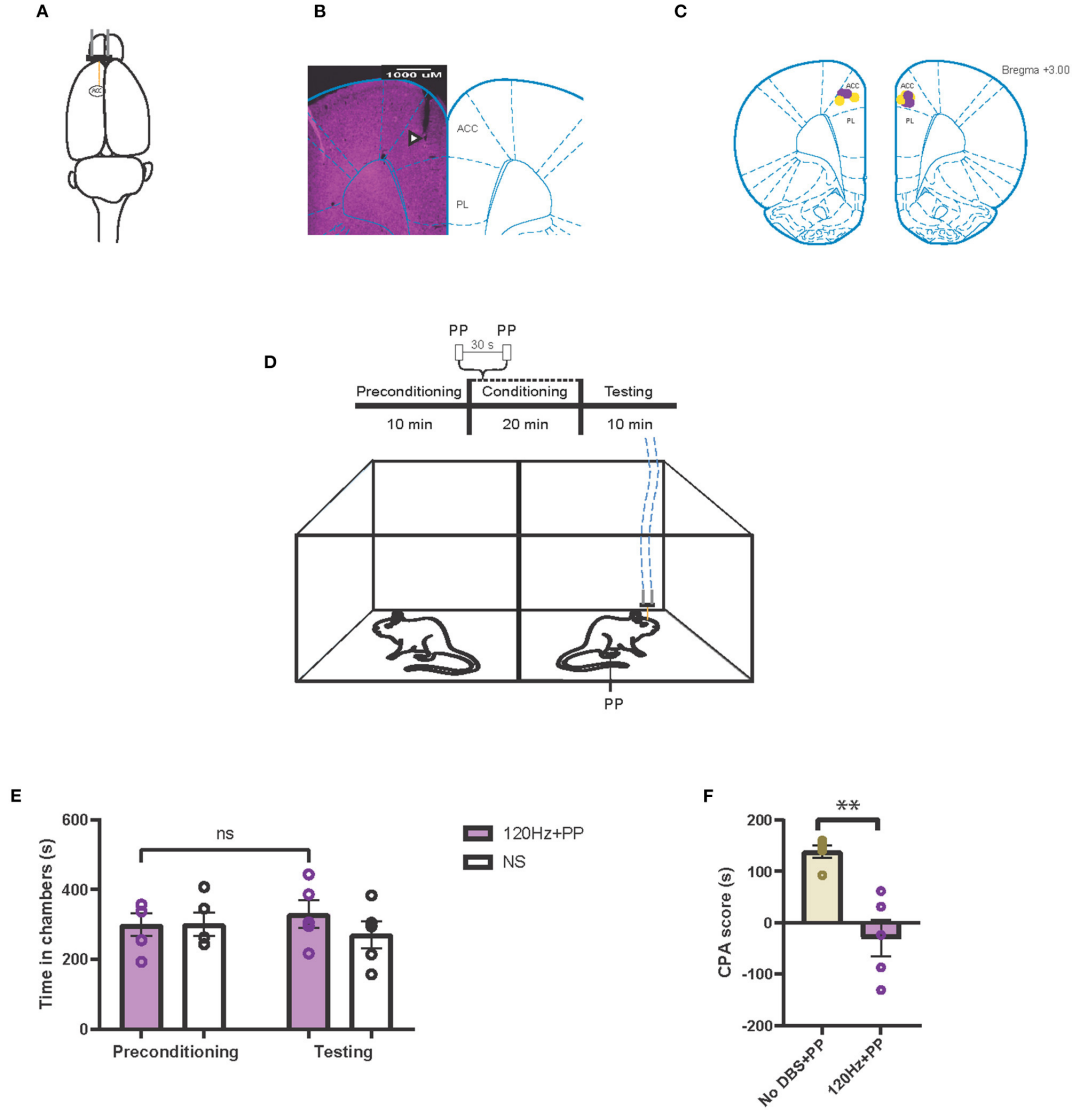
High-frequency neurostimulation of the ACC decreases pain aversive response. **(A)** Stimulating electrode (orange) implanted in the left anterior cingulate cortex (ACC). **(B)** Histology verifying the electrode implantation in the ACC. White triangle indicates location of the electrode. **(C)** Schematic showing the intracranial electrode sites in the ACC. Yellow dots denote the CPA control group implantations. Purple dots denote the CPA experimental group implantations. **(D)** Schematic of the CPA assay to test pain aversion under high-frequency stimulation of ACC. Aversive response was triggered by a noxious mechanical stimulus (PP) applied to the hind paws 30 s apart. In the control chamber, neither mechanical (NS) nor electrical stimulus were given; in the treatment chamber, the mechanical stimulus was paired with 120-Hz electrical stimulation to the ACC, denoted by the blue dashed line. **(E)** After conditioning, rats receiving noxious mechanical stimulation (PP) paired with 120-Hz electrical stimulation to the ACC did not develop an aversion to the treatment chamber. *n* = 5; NS, *P* = 0.4503, paired *t* test. Data are presented as mean ± s.e.m. **(F)** CPA scores for rats given noxious mechanical stimulation (PP) paired with sham electrical stimulation (yellow) compared to rats that were given PP paired with 120-Hz electrical stimulation (purple) to the ACC. *n* = 5; ^**^P = 0.0021, unpaired *t*-test. Data are presented as mean ± s.e.m.

In addition to the PFC, the ACC has long been known to process the affective or aversive component of pain and thus constitutes another important neuromodulation target for pain, particularly for the pain-aversive experience (33, 49). Thus, we investigated the effect of both low- and high-frequency stimulation of the ACC on pain aversion (Figures 4A–C). First, we tested the effect of high-frequency neuromodulation of the ACC on the pain-aversive response using a CPA assay. During conditioning, one chamber was paired with a peripheral noxious stimulus as well as with 120-Hz cortical stimulation, whereas the opposite chamber was paired with neither peripheral noxious nor cortical stimulation (Figure 4D). During the testing phase, rats did not exhibit an avoidance for the chamber paired with the noxious stimulus (Figure 4E). In contrast, sham cortical stimulation did not prevent the avoidance of the chamber paired with the peripheral noxious stimulus (Figure 2B). We compared the CPA scores for the sham stimulation and the 120-Hz stimulation and found that high-frequency stimulation indeed reduced the aversive pain response produced by mechanical noxious stimuli (Figure 4F).

**Figure 5:**
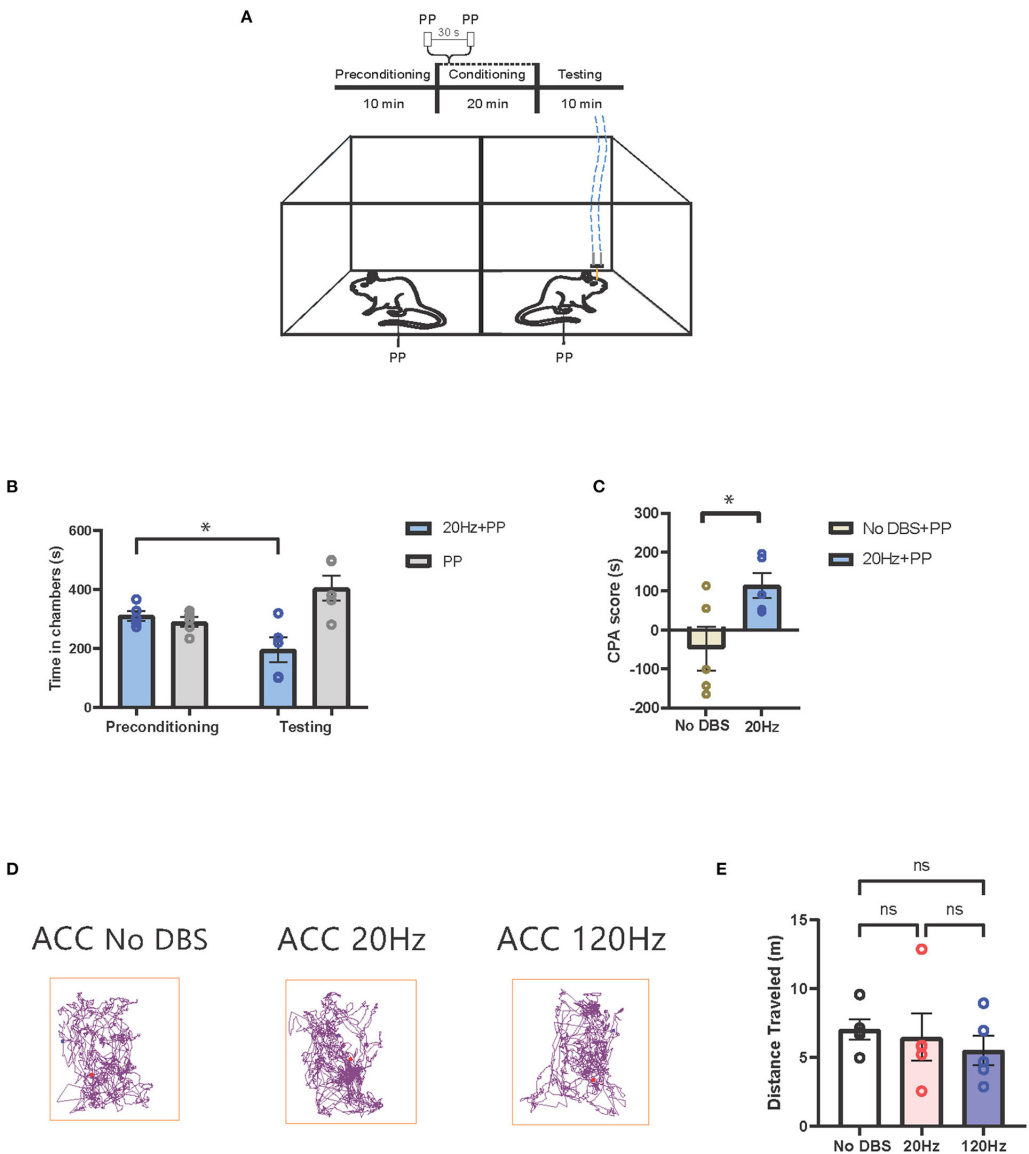
Low-frequency neurostimulation of the ACC increases pain aversive response. **(A)** Schematic of the CPA assay to test pain aversion under activation of ACC. Aversive response was triggered by a noxious mechanical stimulus (PP) applied to the hind paws 30 s apart in both chambers. In the control chamber, no electrical stimulation was given; in the treatment chamber, the mechanical stimulus was paired with 20-Hz electrical stimulation to the ACC, denoted by the blue dashed line. **(B)** After conditioning, rats receiving noxious mechanical stimulation (PP) paired with 20-Hz electrical stimulation to the ACC developed an aversion to the treatment chamber. *n* = 5; ^*^P = 0.0235, paired *t*-test. Data are presented as mean ± s.e.m. **(C)** CPA scores for rats given noxious mechanical stimulation (PP) paired with sham stimulation (yellow) compared to rats that were given PP paired with 20-Hz electrical stimulation (blue) to the ACC. *n* = 5; ^*^P = 0.0356, unpaired *t-*test. Data are presented as mean ± s.e.m. **(D)** Locomotion of the rat within a chamber recorded by AnyMaze when given electrical stimulation at different frequencies (No DBS, 20 and 120 Hz) to the ACC. The purple line denotes the path taken; the red dot marks the origin. The frequency of the electrical stimulation administered had no noticeable effects on locomotion. **(E)** The frequency of electrical stimulation to the ACC had no significant effect on locomotion. *n* = 5; No DBS vs. 20 Hz, *P* = 0.9476; No DBS vs. 120 Hz, *P* = 0.5295; 20 Hz vs. 120 Hz, *P* = 0.8261; one-way ANOVA, Tukey's multiple comparisons test with repeated measures. Data are presented as mean ± s.e.m.

Next, we tested the effects of low-frequency stimulation of the ACC on the pain aversive response. During conditioning, we provided pain stimulus to both chambers, paired with no electrical stimulation in one chamber, and 20-Hz low-frequency stimulation in the opposite chamber (Figure 5A). The rats avoided the chamber associated low-frequency ACC stimulation (Figure 5B). In contrast, sham cortical stimulation paired with PP did not induce the avoidance of the chamber (Figure 3B). Twenty hertz treatment increased the CPA score compared to sham stimulation, suggesting that low-frequency neurostimulation to the ACC increased the pain aversive response (Figure 5C). To confirm the behavioral specificity of electrical stimulation to the ACC, we observed the locomotion of the rats under low-, high-, and sham stimulation conditions and found no changes in locomotive behavior (Figures 5D,E).

## Discussion

In this study, we examined the effect of electrical stimulation to the PL-PFC and ACC for pain modulation in awake, freely moving rats. We found that low-frequency electrical stimulation of the PFC can inhibit sensory and affective components of acute pain, similar to results of previous optogenetic studies using stimulation at the same frequencies (7, 21). In addition, we observed that high-frequency electrical stimulation of the ACC can reduce the affective components of acute pain. These results demonstrate the feasibility of the PFC and ACC as potential neuromodulation targets for clinical pain management.

An interesting finding in our study is that lower-frequency stimulations of the PL-PFC reduced pain, whereas stimulation at a very high frequency actually made pain worse. One possible explanation is that such frequency dependent effects may be due to activation or inhibition of local circuitry. Prior studies employing optogenetics have shown that at 20-Hz, excitatory neurons could be activated in the PL-PFC to reduce pain (7, 21). Unlike optogenetic stimulation which can employ transgenes or viral vectors to target specific types of excitatory or inhibitory cells (50), electrical stimulation does not directly target specific classes of neurons. Comparing our results with data from prior optogenetic studies, however, it is possible that at lower frequencies, electrical stimulation produces a net activation of excitatory neurons. Meanwhile, it has been shown that very high frequency (200 Hz) stimulation may have an inhibitory effect on targeted brain regions; this strategy has been used for the treatment of Parkinson's disease (70). Thus, relatively higher frequency (120 Hz) stimulation of the PL-PFC in our study may produce a net activation of inhibitory neurons to produce an overall inhibitory effect. Future studies using simultaneous administration of electrical stimulation and/or pharmacological, optogenetic, chemogenetic modulation, in combination with either neuroimaging or neural recordings may reveal details on cellular and circuit mechanisms of electrical stimulation.

In this study, in order to facilitate clinical translation, we stimulated the PFC and ACC unilaterally. An earlier study also showed that ipsilateral PFC stimulation had similar anti-nociceptive effects as bilateral stimulation (22). However, future studies may be needed to further characterize the laterality of electrical stimulation of the PFC or ACC for pain relief. Furthermore, we have focused on the frequency-dependent effect of electrical stimulation in our present study; meanwhile, a range of stimulation parameters, such as different amplitudes or stimulation patterns (continuous vs. burst stimulation), could be tested to further advance clinical translation.

While we studied the PFC and ACC, additional cortical and subcortical regions, such as the motor cortex, have already been shown to be analgesic targets for neuromodulation (38, 39, 51, 52). Currently, in the US, clinical neuromodulation as a treatment for pain has been limited to continuous spinal cord stimulation, typically administered at mid-frequencies around 40–60 Hz (53). However, electrical stimulation targeting brain regions relevant to pain-processing, such as the motor cortex, sensory thalamus, periacqueductal gray, and nucleus accumbens have been studied as potential therapies as well (35, 36, 38, 39, 41–46, 51, 52, 54–69). High-frequency stimulations similar to those administered for deep brain stimulation (DBS) treatment of Parkinson's disease have been utilized to inhibit neural activity in pain-producing brain regions (70). Other studies have used low-frequency direct stimulation in an attempt to activate target brain regions. Our results here reinforce the concept that cortical structures, similar to subcortical structures, can be targeted by neurostimulation techniques to produce pain relief.

Although we have shown that electrical stimulation can successfully modulate pain, the non-specific nature of this technique remains a limitation. Like many other brain areas, the ACC and PFC have multiple functions. Since there is not a single target for pain modulation, neuromodulation techniques such as electrical stimulation can be expected to have non-specific effects, similar to what is found with deep brain stimulation treatments for neuropsychiatric diseases (71, 72). Another risk associated with electrical stimulation of cortical regions is its potential to induce seizures when administered at high-frequencies and intensities for a prolonged period of time (73). To improve pain treatment specificity, one option is demand-based or closed-loop electrical stimulation. Such technology, however, requires real-time pain decoding, which continues to be a challenge in the pain field. Another solution is to adjust stimulation parameters to combine stimulation across multiple pain-processing regions.

In conclusion, we have demonstrated that electrical stimulation of the PFC or ACC can effectively produce pain relief depending on the stimulation frequency. Our results indicate that frequency-specific cortical stimulation can be expected to target both sensory and affective pain symptoms. By characterizing the frequency dependence of electrical stimulation, our study can contribute to future development of this technique to become a useful tool in the treatment of pain.

## Data Availability Statement

The original contributions presented in the study are included in the article/Supplementary Material, further inquiries can be directed to the corresponding author/s.

## Ethics Statement

The animal study was reviewed and approved by The Institutional Animal Care and Use Committee (IACUC) of the New York University School of Medicine (NYUSOM).

## Author Contributions

JW, YL, and QZ conceived and designed the experiments. YL, HX, GS, BV, and HJ performed the experiments and collected the data. YL and HX performed statistical analysis. QZ and JW wrote the manuscript with help from other authors.

## Conflict of Interest

The authors declare that the research was conducted in the absence of any commercial or financial relationships that could be construed as a potential conflict of interest.

## Publisher's Note

All claims expressed in this article are solely those of the authors and do not necessarily represent those of their affiliated organizations, or those of the publisher, the editors and the reviewers. Any product that may be evaluated in this article, or claim that may be made by its manufacturer, is not guaranteed or endorsed by the publisher.

## Supplementary Material

Supplementary Figure 1 **(A)** Schematic of the Hargreaves test (IR 70). Thermal stimulation from the infrared (IR) emitter was applied to the rat's hind paw, contralateral to the electrode implanted within the PL-PFC. Electrical stimulation was delivered to the PL-PFC concurrent with onset of the thermal stimulation. **(B)** Application of low frequency (2 Hz) electrical stimulation of the PL-PFC prolonged the withdraw latency relative to that of the baseline (0 Hz), in which no electrical stimulation was given. 20 Hz also prolonged the withdrawal latency and proved more effective in providing pain relief. *n* = 6 rats. No DBS vs. 2 Hz, ^*^*P* = 0.0193; No DBS vs. 20 Hz, ^**^*P* = 0.0052; 2 vs. 20 Hz, ^*^*P* = 0.0186; one-way ANOVA, Tukey's multiple comparisons test with repeated measures. Data are presented as mean ± s.e.m. **(C)** Schematic of the CPA assay to test pain aversion under modulation of PL-PFC. In both chambers, aversive response was triggered by a noxious mechanical stimulus (pinprick, PP) applied to the hind paws 30 s apart. One of the chambers received 2-Hz electrical stimulation, and the opposite chamber was paired with electrical stimulation at 20 Hz. The dashed blue line denotes electrical stimulation. **(D)** After conditioning, rats avoided the chamber paired with 2-Hz electrical stimulation to the PL-PFC, when presented with PP. *n* = 5; ^*^*P* = 0.0123, paired *t* test. Data are presented as mean ± s.e.m. **(E)** Schematic of the CPA assay to test pain aversion under modulation of PL-PFC. Both chambers received PP, administered at intervals of 30 s. One of the chambers received 20-Hz electrical stimulation, and the opposite chamber was paired with electrical stimulation at 120 Hz. The dashed blue line denotes electrical stimulation. **(F)** After conditioning, rats avoided the chamber paired with 120-Hz electrical stimulation to the PL-PFC, when presented with PP. *n* = 5; ^**^*P* = 0.0072, paired *t* test. Data are presented as mean ± s.e.m. **(G)** Schematic of the CPA assay to test aversion under high-frequency (120 Hz) modulation of PL-PFC. In a two-chamber set-up, one of the chambers received sham stimulation (No DBS), and the opposite chamber was paired with electrical stimulation at 120 Hz. Rats in both chambers did not receive any noxious peripheral mechanical stimulus (NS). The dashed blue line denotes electrical stimulation. **(H)** After conditioning, rats did not show aversion to either chamber. *n* = 5; *P* = 0.9625, paired *t-*test. Data are presented as mean ± s.e.m.
