# Supplementary figures and images for: Frequency Dependent Electrical Stimulation of PFC and ACC for Acute Pain Treatment in Rats
Source: Front Pain Res (Lausanne). 2021 Aug 27;2:728045. doi: 10.3389/fpain.2021.728045 (PMC8915567; doi:10.3389/fpain.2021.728045)

(A)

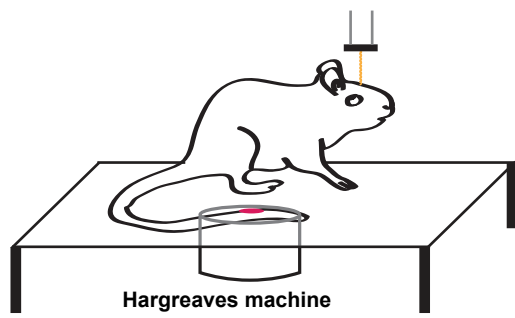

(B)

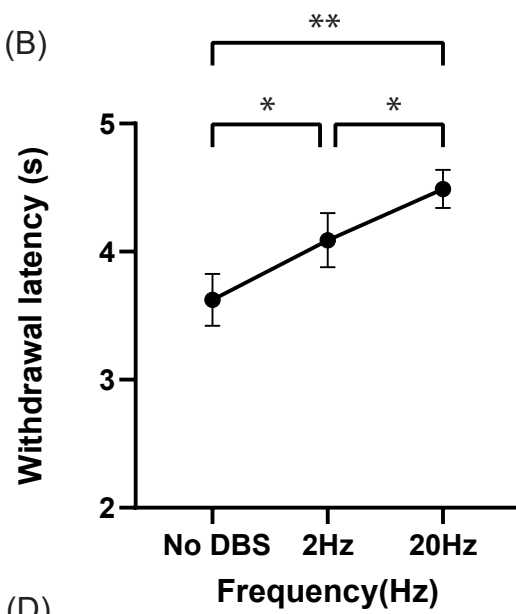

(C)

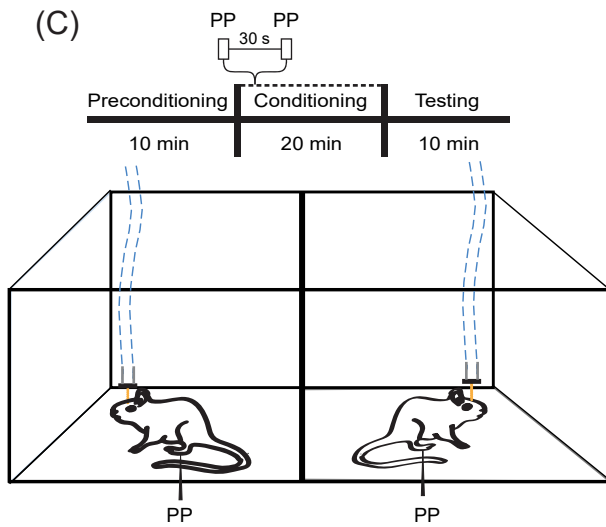

(D)

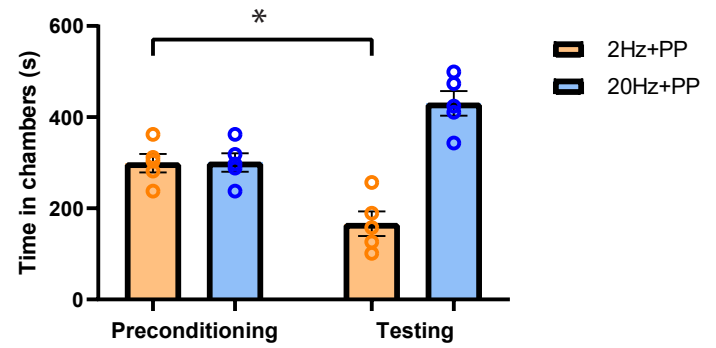

(E)

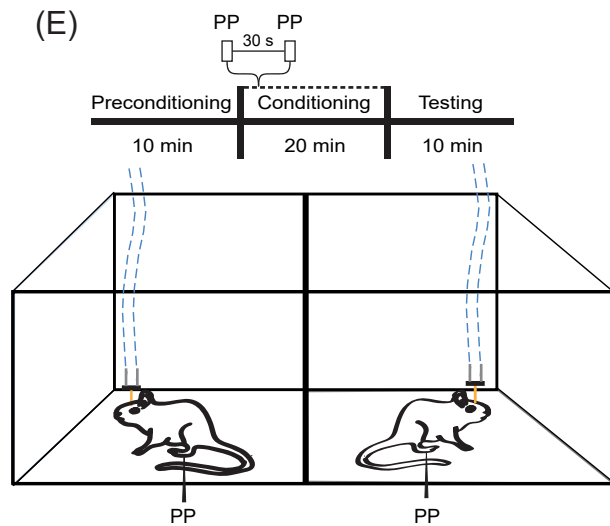

(F)

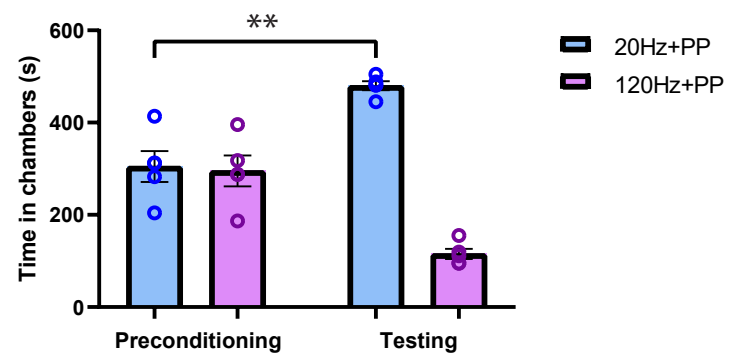

(G)

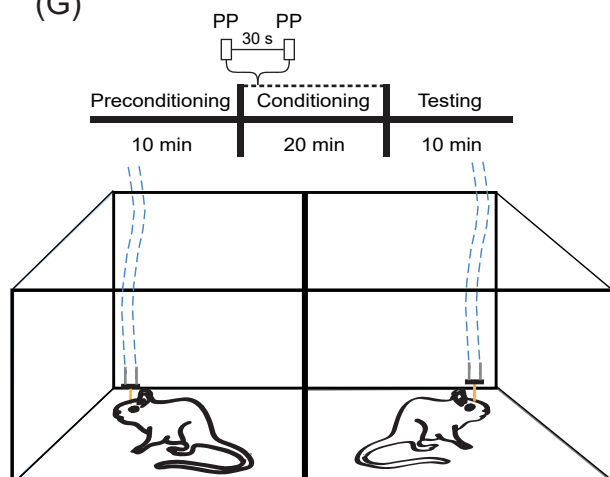

(H)

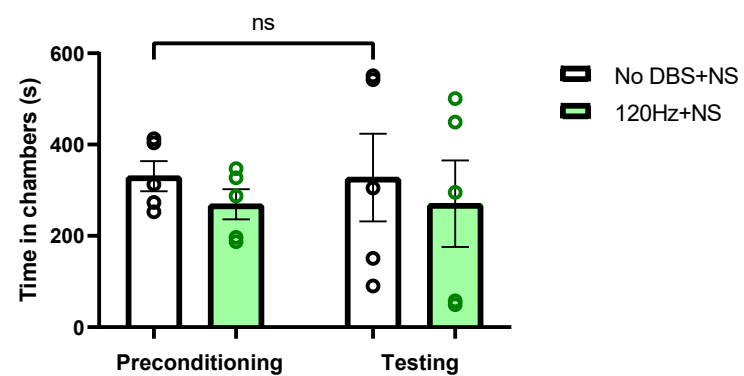

Supplement: Supplementary Figure 1 — (A) Schematic of the Hargreaves test (IR 70). Thermal stimulation from the infrared (IR) emitter was applied to the rat's hind paw, contralateral to the electrode implanted within the PL-PFC. Electrical stimulation was delivered to the PL-PFC concurrent with onset of the thermal stimulation. (B) Application of low frequency (2 Hz) electrical stimulation of the PL-PFC prolonged the withdraw latency relative to that of the baseline (0 Hz), in which no electrical stimulation was given. 20 Hz also prolonged the withdrawal latency and proved more effective in providing pain relief. n = 6 rats. No DBS vs. 2 Hz, *P = 0.0193; No DBS vs. 20 Hz, **P = 0.0052; 2 vs. 20 Hz, *P = 0.0186; one-way ANOVA, Tukey's multiple comparisons test with repeated measures. Data are presented as mean ± s.e.m. (C) Schematic of the CPA assay to test pain aversion under modulation of PL-PFC. In both chambers, aversive response was triggered by a noxious mechanical stimulus (pinprick, PP) applied to the hind paws 30 s apart. One of the chambers received 2-Hz electrical stimulation, and the opposite chamber was paired with electrical stimulation at 20 Hz. The dashed blue line denotes electrical stimulation. (D) After conditioning, rats avoided the chamber paired with 2-Hz electrical stimulation to the PL-PFC, when presented with PP. n = 5; *P = 0.0123, paired t test. Data are presented as mean ± s.e.m. (E) Schematic of the CPA assay to test pain aversion under modulation of PL-PFC. Both chambers received PP, administered at intervals of 30 s. One of the chambers received 20-Hz electrical stimulation, and the opposite chamber was paired with electrical stimulation at 120 Hz. The dashed blue line denotes electrical stimulation. (F) After conditioning, rats avoided the chamber paired with 120-Hz electrical stimulation to the PL-PFC, when presented with PP. n = 5; **P = 0.0072, paired t test. Data are presented as mean ± s.e.m. (G) Schematic of the CPA assay to test aversion under high-frequency (120 H [file Data_Sheet_1.PDF]
